# PROTOCOL: The FRIENDS preventive programme for reducing anxiety symptoms in children and adolescents: A systematic review

**DOI:** 10.1002/cl2.1374

**Published:** 2023-12-15

**Authors:** Trine Filges, Geir Smedslund, Tine Eriksen, Kirsten Birkefoss

**Affiliations:** ^1^ VIVE – The Danish Center for Social Science Research Copenhagen Denmark; ^2^ Norwegian Institute of Public Health Oslo Norway; ^3^ VIVE – The Danish Center for Social Science Research Aarhus Denmark

## Abstract

This is the protocol for a Campbell systematic review. The objectives are as follows. The main objective of this review is to answer the following research question: What are the effects of the FRIENDS preventive programme on anxiety symptoms in children and adolescents? Further, the review will attempt to answer if the effects differ between participant age groups, participant socio‐economic status, type of prevention (universal, selective or indicated), type of provider (lay or mental health provider), country of implementation (Australia or other countries) and implementation issues in relation to the booster sessions and parent sessions (implemented, partly implemented or not at all).

## 1 BACKGROUND

### 1.1 The problem, condition or issue

An estimated one in eight (12.7%) children and youth aged 4–18 years from high‐income countries (as classified by the World Bank, 2021 [World Bank Group, 2021]) have mental disorders at any given time, causing symptoms and impairment, therefore requiring treatment (Barican et al., 2022). Anxiety disorders are amongst the most common psychiatric disorders, occurring in 5.2% of all children and youth aged 4–18 years from high‐income countries (Barican et al., 2022).

Every child and adolescent faces normal, developmentally appropriate worries, fears, and shyness. For example, primary school‐age children commonly have worries about injury and natural events, whereas older children and adolescents typically have worries and fears related to school performance, social competence, and health issues (Beesdo et al., 2009). Pathological anxiety significantly, however, interferes with a child's ability to handle a wide variety of everyday activities, such as interpersonal relationships, social competence, peer relations and school adjustment. If left untreated, childhood anxiety may develop over the years into a chronic adult anxiety disorder or, in some cases, clinical depression (Barrett & May, 2007). Studies show that most youths who experience psychological distress do not seek professional help (Biddle et al., 2004), and youths with a mental disorder are less likely to use mental health services than adults (Mack et al., 2014). It is estimated that less than 25% of all children and youths with an anxiety disorder receive professional help (Merikangas et al., 2011; Wang et al., 2007). In addition, there is often a delay of 9–23 years from onset to first treatment for the disorder (Wang et al., 2007). For these reasons, it is important to prevent that children and youth with elevated anxiety symptoms move on to fully develop anxiety disorders.

As anxiety, fear, and stress responses are often considered normative experiences, children and adolescents may benefit from anxiety prevention programmes regardless of risk status.

An anxiety prevention programme that is manualised, well‐structured, and can be easily integrated into school curriculums is the FRIENDS programme. FRIENDS is based on a firm theoretical model which addresses cognitive, physiological and behavioural processes that are seen to interact in the development, maintenance and experience of anxiety (Barrett & May, 2007). FRIENDS is an acronym for the skills taught throughout the programme: · Feelings. · Remember to Relax. Have quiet time. · I can do it! I can try (Inner helpful thoughts) · Explore Solutions and Coping Step Plans. · Now reward yourself! You've done your best! · Don't forget to practice. · Smile! Stay calm, Stay Strong and talk to your support networks!

In a meta‐analysis of anxiety prevention programmes, Fisak et al. (2011) found FRIENDS to be more effective for anxiety reduction than other prevention programmes. The vast majority of the studies included in the review were performed in Australia; 8 of the 10 evaluations on FRIENDS were performed in Australia. As noted by the review authors, more research is needed to determine the degree to which the effectiveness of the programme is generalisable to nations other than Australia. Since this review was carried out, several trials on the effectiveness on FRIENDS have been carried out in countries other than Australia, and it may now be possible to answer the question on effectiveness outside of Australia.

### 1.2 The intervention

The FRIENDS programme is a 10‐session manualised cognitive behavioural therapy (CBT) programme which can be used as both prevention and treatment of child and youth anxiety (Barrett et al., 2014).

The FRIENDS protocol has been adapted into three developmentally‐sensitive programmes:

- Fun FRIENDS (4–7 years)
- FRIENDS for life (8–11 years)
- My FRIENDS Youth (12–16 years)

However, note that these three age‐appropriate programmes and their titles are the current versions of the FRIENDS programme. The FRIENDS programme was developed by Dr. Barrett in 1998 (a refinement of the programme ‘*Coping Koala*’ to reflect a user‐friendly early intervention and prevention format), and was expanded into two parallel age groups – FRIENDS *for Children* 7–11 years, and FRIENDS *for Youth* 12–16 years.

A new general title for the programme, ‘FRIENDS for Life’ was introduced in 2005 (Barrett & May, 2007) and a developmentally tailored, downward extension of the two pre‐existing FRIENDS for Life programmes was added (the Fun FRIENDS programme, Pahl & Barrett, 2007). Today the FRIENDS programme is broken up into the three age groups shown above (in addition there is a version aimed at adults aged 16+ which is not included in this review). Each of these developmentally tailored programmes is structured and implemented in the same way (Higgins & O'Sullivan, 2015). FRIENDS is a manual based programme which consists of 10 1‐h lessons (although the programme allows for flexible roll‐out as long as the sequence of the sessions are maintained) plus two follow‐up booster sessions, during which the key cognitions and behaviours associated with anxiety are targeted and addressed. The programmes also involve a parent component which consists of parent psycho‐educational sessions where parents are helped to understand anxiety, develop appropriate strategies to deal with their own anxiety, if necessary, and improve their child management and problem‐solving skills. Certification is required for all professionals who want to use the FRIENDS programme. Certified professionals further have to be re‐certified every third year to ensure that they are updated with the latest developments of the programme. FREINDS can be run by teachers or mental health care professionals, and it can be run as a whole class programme, or as a small group intervention.

The intervention of interest is the preventive anxiety programme FRIENDS (the three age‐appropriate programmes). The comparison population are children and adolescent who do not participate in FRIENDS programmes.

As recommend by the Institute of Medicine Report (Mrazek & Haggerty, 1994), and the updated report (O'Connell et al., 2009) we will define prevention as those interventions that occur before the onset of a clinically diagnosed disorder. Although the programme has been designed to be effective as both a treatment and a prevention course (mostly school‐based), we will only include preventive programmes.

The type of prevention may be universal or targeted. The Institute of Medicine report published in 1994, categorised prevention programmes based on the population targeted (Mrazek & Haggerty, 1994). Specifically, universal prevention programmes are applied to the general population, without focusing on the risk status. Selective programmes target those (individuals or groups) who are identified as exhibiting an elevated risk for developing a disorder based on established risk factors (e.g., socio‐economic status, Barrett et al., 2017), and indicated programmes target those (individuals) who exhibit problematic behaviours predicting a high level of risk or initial symptoms of a disorder but who do not yet meet criteria for the disorder. In general, the type of prevention programme utilised (i.e., universal, selective or indicated) may be a crucial methodological factor associated with programme effectiveness (Donovan & Spence, 2000). All types of preventive programmes are eligible whereas treatment programmes (for those with a diagnosis and in need of treatment) are not eligible.

All types of providers (e.g., teachers, mental health providers) and all types of settings (e.g., school based, community based) will be eligible.

### 1.3 How the intervention might work

FRIENDS programmes are a suite of programmes (including Fun FRIENDS, FRIENDS for Life and FRIENDS for Youth), which aim to improve resilience (or coping) skills in children and youth and reduce anxiety and improve mental health and wellbeing.

The programme is based on CBT and positive psychology and uses a play‐based and experiential learning approach to provide cognitive behavioural skills in a developmentally appropriate manner. During each session children and youth are taught skills, aimed at helping them to increase their coping skills through stories, games, videos and activities.

**Figure 1:**
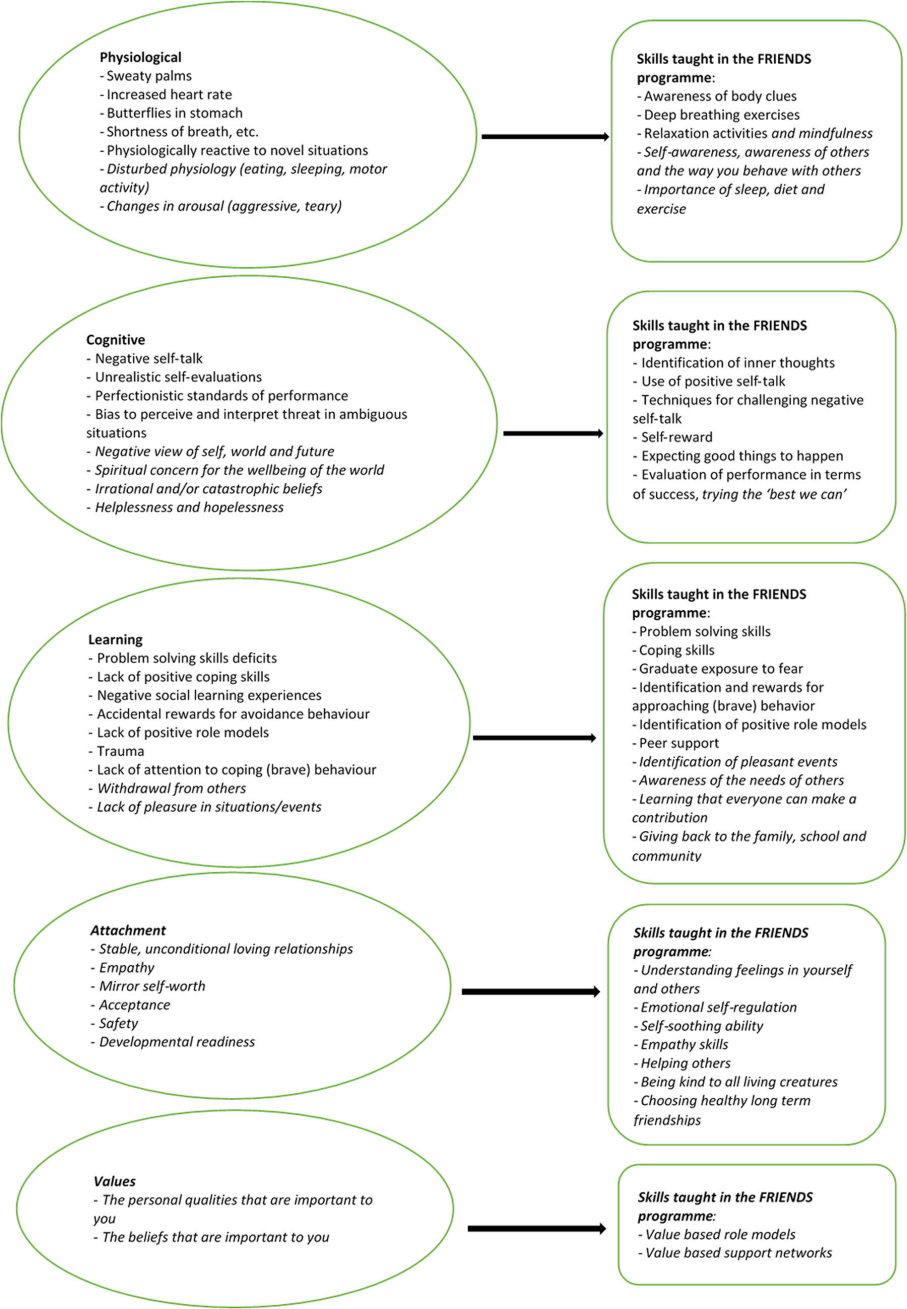
How the intervention works.

The theoretical model for the prevention and early intervention for anxiety in specific relation to FRIENDS for Life is shown in Figure 1 (based on Barrett, 1999, 2020; Wigelsworth et al., 2018). The programme has gone through various updates since 1998. The figure therefore shows the original content as reported in Barrett (1999) and the additions in *cursive* as reported in Wigelsworth et al. (2018) and Barrett (2020).

The programme addresses child and youth anxiety by focusing on (a) physiological or body reactions: the physical reactions our bodies experience when we are feeling worried, nervous, or afraid; (b) cognitive or ‘mind’ processes: the inner thoughts we have about ourselves, others, and situations; (c) learning or behavioural skills: the acquisition of now skills to cope with and manage anxiety; (d) attachment: stable, unconditional loving relationships; and (e) values: identify personal qualities important to you. The programme aims to teach coping skills such as understanding and managing emotions to assist children and youth in responding to uncomfortable emotions in appropriate and helpful ways. The coping skills increase the children's (and youth's) resilience and protects them from developing anxiety.

### 1.4 Why it is important to do this review

#### 1.4.1 Policy relevance

Concerns and worries are a normal part of the everyday life of children and adolescents (Weems & Stickle, 2005). Childhood fears such as worries about loss or separation from parents or worries about personal injury, death and natural disasters are often a normal part of childhood development (Warren & Sroufe, 2004). However, as Warren and Sroufe (2004) argue, these worries can become problematic if they become persistent, frequent and severe enough to interfere with or limit the child's everyday life and functioning. There are therefore good reasons for increased focus on anxiety prevention over treatment.

Evidence shows that a high proportion of children do not grow out of their anxiety disorders during adolescence and adulthood (Majcher & Pollack, 1996). Anxiety disorders are amongst the most prevalent psychiatric disorders, with a prevalence rate of 10.4% in Western European, North American and Australasian populations (Baxter et al., 2012). According to research by Thompson et al. (2004, 2008) most sufferers of anxiety do not access treatment until well into adulthood and even those who do access appropriate help typically suffer for many years before receiving that help. It is therefore important to prevent that children and youth with elevated anxiety symptoms move on to fully develop anxiety disorders.

#### 1.4.2 Other reviews

We have located three systematic reviews on anxiety preventive programmes for children and adolescents in general (including FRIENDS) and three reviews on the specific programme FRIENDS.

Werner‐Seidler et al. (2017) and the update Werner‐Seidler et al. (2021):

Performed a systematic review on school‐based depression and anxiety prevention programmes for young people (i.e., children or adolescents with a mean age between 5 and 19 years met their inclusion criteria). In the update the authors searched up to October 2020. Of the 118 included studies, 34 studies were classified as focused on preventing anxiety, and 30 were classified as being mixed depression/anxiety prevention programmes. A total of 18 evaluations of the FRIENDS anxiety prevention programme were included (and 4 follow‐up studies), the majority (15) were classified as anxiety prevention programmes, but 3 were classified as mixed depression/anxiety prevention programmes. The classification of the focus of the studies, however, plays no role in the analyses as all studies reporting anxiety outcomes (72) were used in the analyses of anxiety regardless of the focus of the study as classified by the review authors. Separate meta‐analyses were performed for depression and anxiety outcomes. Single factor subgroup analyses and multiple meta‐regressions were performed with a number of study characteristics entered as predictors of the outcomes. In particular programme content was examined, but the impact of FRIENDS was not investigated, rather the impact of CBT‐based programmes vs other therapeutic approaches was investigated. None of the effect sizes analysed were corrected for clustering at neither the school nor the class level (if needed). As the review included both universal prevention programmes, which typically are both assigned and delivered at the class or school level and targeted (selective/indicative) prevention programmes which may be assigned at the school or class level but is not delivered to whole classes, this may have biased the results of the meta‐analyses considerably.

Ahlen et al. (2015):

Performed a systematic review on universal prevention programmes targeting anxiety or/and depression in school‐aged children 6–18 years. The searches were performed in July 2012. Of the 30 included studies, the primary aim of the intervention was to prevent depression in 13 studies, to prevent anxiety in 10 studies, and to prevent both anxiety and depression in 7.

A total of six evaluations of the FRIENDS anxiety prevention programme were included (and two follow‐up studies), all were classified as anxiety prevention programmes.

All studies reporting anxiety outcomes (18) were used in the analyses of anxiety regardless of the primary aim of the intervention as classified by the review authors. Separate meta‐analyses were performed for depression and anxiety outcomes. The intervention ‘FRIENDS for life’ was examined as moderator for anxiety symptoms using single factor subgroups. No significant differences were found.

Higgins and O'Sullivan (2015).

The review provided a narrative summary of five randomised controlled trials (and two follow‐up studies) which examined the effectiveness of the FRIENDS programme as a preventative universal intervention for child and youth (aged 4–16 years) anxiety. Studies published in peer‐reviewed journals between 2000 and 2013 were eligible.

Maggin and Johnson (2014).

This is a systematic review with meta‐analysis on school based FRIENDS for students enroled in Kindergarten to grade 12. Only group based experimental or quasi experimental studies with a control group reporting on standardised measures of anxiety were eligible. The review included 17 studies (reported in 16 manuscripts) of which 2 were follow‐up studies.

The final search date (reported in Maggin & Johnson, 2019, which was a reply to a critique provided in Barrett et al., 2017) was October 2010. All analyses in the review were separated by the review authors' definition of low‐risk students and students with elevated risk (defined as pre‐test scores within the clinical range, though the cut‐off scores used were not reported).

There are further a number of other ambiguities. First, it is unclear which studies provide effect estimates to which analyses (low/high risk students and post/follow up) as the numbers available (according to table 1) do not add up to the numbers reported used in table 4. Second, it is unclear which anxiety measures are used. Five studies reported results for two or more standardised anxiety measures. The review authors stated that they randomly selected one measure from each of these studies to include in the meta‐analyses. It was not reported which ones were used. Last, it was reported that ‘a series of moderator analyses of student characteristics and programme features failed to predict treatment outcomes’ (p. 295), however, the analyses were not shown, and it was not reported which student characteristics and programme features were used nor which model was used other than ‘a mixed effects framework’ was used.

Fisak et al. (2011).

The purpose of this review was to provide a comprehensive review of the effectiveness of child and adolescent (below the age of 18) anxiety prevention programmes, including universal as well as targeted (selective/indicative) programmes. Programmes in which depression or general stress management was the primary goal, and where anxiety was only measured as a secondary variable, were not eligible. The date of database search is not reported, but it is reported that the hand searches (of selected journals) were performed from 1970 to the end of 2009. A total of 30 studies (of which 4 were follow‐up studies to previously published studies) with a comparison group was found of which 10 were evaluating the FRIENDS intervention. Based on moderator analyses, it was found that studies utilising the FRIENDS programme were more effective than programmes not utilising FRIENDS. However, as noted by the authors, more research is needed to determine the degree to which the effectiveness of the programme is generalisable to nations other than Australia (8 of the 10 evaluations on FRIENDS were performed in Australia). None of the effect sizes analysed were corrected for clustering at neither the school nor the class level (if needed). As the review included both universal prevention programmes and targeted (selective/indicative) prevention programmes this may have biased the results of the meta‐analyses considerably.

Briesch et al. (2010).

This was a report on a literature search, conducted to identify all empirical studies of the FRIENDS programme published in peer‐reviewed journals. The search was not described nor documented other than that they screened the list of research abstracts provided on the programme developers' website (no web address was reported), and conducted literature searches using the PsycINFO and MEDLINE databases. No search terms or date was reported. The review cannot be labelled systematic and further no meta‐analysis was performed. The authors reported the range of effect sizes in the studies (14 studies were found) and an average (simple average) effect size but no other statistics such as standard errors, confidence intervals or *p*‐values.

We specifically searched the Cochrane Library for Cochrane systematic reviews and located one marginally relevant for the current review. James et al. (2005) and the updates James et al. (2015) and James et al. (2020), examined the effect of CBT treatment interventions for childhood anxiety disorders. Eligible participants were children and adolescents younger than age 19, who met diagnostic criteria for an anxiety disorder diagnosis. Primary outcomes were remission of primary anxiety diagnosis post‐treatment and number of participants lost to post‐treatment assessment. Secondary outcomes included remission of all anxiety diagnoses, reduction in anxiety symptoms and depressive symptoms and improvements in global functioning. In the most updated (James et al., 2020) 87 studies were included of which 5 evaluated FRIENDS. No separate analysis of FRIENDS was provided nor was FRIENDS included as a moderator.

Besides being up‐to‐date, a major difference between these systematic reviews and the current review is that we will focus on the FRIENDS intervention delivered as universal, selective and indicated preventive programmes. We will only include studies with a control group and with participants from at least two units (e.g., school or class) in each of the groups (treatment and control). All relevant outcome areas will be analysed separately in a meta‐analysis taking into consideration the unit of analysis (cluster or individual) and the dependencies between effect sizes. We will be transparent concerning which studies and which measures are used in each analysis. In addition, the specific data used for any cluster correction and any moderator analyses will be reported in detail.

## 2 OBJECTIVES

The main objective of this review is to answer the following research question: What are the effects of the FRIENDS preventive programme on anxiety symptoms in children and adolescents? Further, the review will attempt to answer if the effects differ between participant age groups, participant socio‐economic status, type of prevention (universal, selective or indicated), type of provider (lay or mental health provider), country of implementation (Australia or other countries) and implementation issues in relation to the booster sessions and parent sessions (implemented, partly implemented or not at all).

## 3 METHODS

### 3.1 Criteria for considering studies for this review

#### 3.1.1 Types of studies

The proposed project will follow standard procedures for conducting systematic reviews using meta‐analysis techniques.

Randomised (and non‐randomised) controlled trials will be included. To summarise what is known about the possible causal effects of programme participation, we will include all study designs that use a control group, that is, a group of children/youth not participating in the intervention. The control group may be offered no treatment or treatment as usual.

The study designs we will include in the review are:

1. Controlled trials (where all parts of the study are prospective, such as identification of participants, assessment of baseline, and allocation to intervention, and which may be randomised or non‐randomised), assessment of outcomes and generation of hypotheses (Higgins & Green, 2011).
2. Non‐randomised studies (attendance in programmes has occurred in the course of usual decisions, the allocation to programmes and no programme is not controlled by the researcher, and there is a comparison of two or more groups of participants, i.e., at least a treated group and a control group).

Studies using single group pre‐post comparisons will not be included. Non‐randomised studies using an instrumental variable approach will not be included—see Supporting Information: Appendix 1 (Justification of exclusion of studies using an instrumental variable (IV) approach) for our rationale for excluding studies of these designs. A further requirement to all types of studies (randomised as well as non‐randomised) is that they are able to identify an intervention effect. Studies where, for example, the treatment is offered to children in one unit (e.g., school or class) only and the comparison group is children at another unit (school/class or more schools/classes for that matter) cannot separate the treatment effect from the unit of school/class effect.

#### 3.1.2 Types of participants

The review will include children and adolescents aged 4 to 16 years who do not meet diagnostic criteria for an anxiety disorder diagnosis. We anticipate that the three age‐appropriate programmes will be provided to children and adolescents in the corresponding age groups; that is, ‘Fun Friends’ to 4–7 year olds, ‘Friends for Life’ to 8–11 year olds and ‘My Friends Youth’ to 12–16 year olds. If studies including participants out‐of‐age range are located they will be eligible if at least 70% of participants are within the age range corresponding to the particular programme or results for a discrete age group within the eligible range is provided. If studies include a mix of children and adolescents with and without a clinically diagnosed anxiety disorder, we will include them if at least 70% of participants are not diagnosed or results of the eligible subgroup (not diagnosed) is provided.

#### 3.1.3 Types of interventions

The intervention of interest is the preventive anxiety programme FRIENDS. Prevention is defined as those interventions that occur before the onset of a clinically diagnosed disorder. Treatment programmes (for those with a diagnosis) are not eligible. The type of prevention may be universal (applied to the general population, without focusing on the risk status), selective (target those (individuals or groups) who are identified as exhibiting an elevated risk for developing a disorder based on established risk factors) or indicated (target those (individuals) who exhibit problematic behaviours predicting a high level of risk or initial symptoms of a disorder but who do not yet meet criteria for the disorder).

The three age‐appropriate preventive anxiety programmes: Fun FRIENDS, FRIENDS for Life (titled FRIENDS for Children before 2005), and My FRIENDS Youth (titled FRIENDS for Youth before 2005) are eligible.

The comparison population are children and adolescent who do not participate in any of the FRIENDS programmes.

#### 3.1.4 Types of outcome measures

The intervention is an anxiety prevention programme and although some studies may report depression outcomes we will limit the analysis to anxiety outcomes. The reason is that an analysis on depression outcomes may be biased as not all studies report depression outcomes, and it cannot be ruled out that those who do have results biased towards a positive effect on depression outcomes.

##### Primary outcomes

The primary focus is on reduction in anxiety symptoms at all time points, measured using psychometrically robust measures of anxiety symptoms that yield symptom scores on continuous scales (Myers & Winters, 2002), such as:

- Child Behavior Checklist‐Anxiety Scale (CBCL‐A) (Achenbach, 1995)
- Multidimensional Anxiety Scale for Children (MASC) (March et al., 1997)
- Revised Children's Anxiety and Depression Scale (RCADS) – Anxiety Scale (Chorpita et al., 2000)
- Revised Children's Manifest Anxiety Scale (RCMAS) (Reynolds & Richmond, 1985)
- Social Anxiety Scale for Adolescents (SAS‐A) (La Greca, 1998)
- Screen for Child Anxiety Related Emotional Disorders (SCARED) (Birmaher, 1999)
- Spence Children's Anxiety Scale (SCAS) (Spence, 1997)
- Social Phobia and Anxiety Inventory for Children (SPAI‐C) (Beidel et al., 1995)
- State‐Trait Anxiety Inventory for Children (STAI‐C) (Spielberger et al., 1973)
- Test Anxiety Scale for Children (TASC) (Sarason, 1975)

These scales could be self‐report or completed by a parent or teacher. Multiple reporters are often used, but the reliability of each reporter is likely to vary with the child's age (Evans et al., 2017). We will therefore analyse reduction in anxiety symptoms separately for (1) self‐reported and (2) parent‐reported or teacher, or both. Multiple measures (on the same reporter, i.e., child, parent or teacher) are also often reported, and we will include the most frequently used measures in the analyses. We will prioritise broad measures of anxiety symptoms (e.g., SCAS, SCARED, MASC, RCMAS, RCADS, CBCL‐A), rather than disorder‐specific symptom measures (e.g., SPAI‐C, SAS‐A, TASC). Effect sizes based on all measures reported in included studies will be reported in the review for transparency, but only one measure per reporter will be included in a particular meta‐analysis as, in our opinion, double counting (of participants) increases precision spuriously, which is inappropriate and unnecessary.

Another primary outcome is prevalence of anxiety diagnosis at medium‐term follow‐up (i.e., between 4 and 12 months) or later. The diagnosis of an anxiety disorder at medium‐term follow‐up or later, must be made by reliable and valid structured interviews for DSM or ICD child and adolescent anxiety disorders, such as:

- Anxiety Disorder Interview Schedule for Children – Child and Parent (ADIS‐C/P) (Silverman, 1987);
- Anxiety Disorder Interview Schedule for Children – Child (ADIS‐C) (Silverman, 1987);
- Anxiety Disorder Interview Schedule for Children – Parent (ADIS‐P) (Silverman, 1987);
- Diagnostic Interview Schedule for Children, Adolescents and Parents (DISCAP) (Holland & Dadds, 1995).

##### Secondary outcomes

As research shows there is a clear relationship between self‐esteem and anxiety (Sowislo, 2013), a secondary focus is on improvement in self‐esteem, measured using psychometrically robust measures of self‐esteem such as:

- Culture‐Free Self‐Esteem Questionnaire (CFSEQ) (Battle, 1992)
- Coping Scale for Children and Youth (CSCY) (Brodzinsky et al., 1992)
- Rosenberg Self Esteem Scale (RSES) (Rosenberg, 1965)
- Self Esteem Inventory (SEI) (Coopersmith, 1989)

If multiple self‐esteem measures/reporters are reported, we include the most frequently used measures in the analysis. Effect sizes based on all measures reported in included studies will be reported.

Any adverse events measured in included studies will be reported.

#### 3.1.5 Duration of follow‐up

Time points for measures considered will be:

- post intervention (including booster sessions)
- short‐term follow‐up (up to 4 months);
- medium‐term follow‐up (4 to 12 months); and
- long‐term follow‐up (over 12 months).

#### 3.1.6 Types of settings

All types of settings (e.g., school based, community based) will be eligible.

### 3.2 Search methods for identification of studies

Relevant studies will be identified through electronic searches in bibliographic databases, grey literature repositories and resources, hand search in specific targeted journals, citation tracking, contact to international experts and Internet search engines. A date restriction of 1998 and onwards will be applied.

#### 3.2.1 Electronic searches

The following electronic bibliographic databases will be searched:

- ERIC (EBSCO)
- Teacher Reference Center
- Academic Search (EBSCO)
- MEDLINE (PubMed)
- Embase (OVID)
- CINAHL (EBSCO)
- Cochrane Library (Cochrane Reviews & Cochrane Central)
- PsycINFO (EBSCO)
- APA PsycNet
- Socindex (EBSCO)
- International Bibliography of the Social Sciences (ProQuest)
- Sociological Abstracts (ProQuest)
- Science Citation Index Expanded (Web Of Science)
- Social Sciences Citation Index (Web Of Science)

We have consulted the list of databases comprised in the article by Kugley (2017) (Kugley, S., Wade, A., Thomas, J., Mahood, Q., Jørgensen, A.‐M.K., Hammerstrøm, K. and Sathe, N. [2017]. Searching for studies: A guide to information retrieval for Campbell systematic reviews. Campbell Systematic Reviews, 13, 1–73. https://doi.org/10.4073/cmg.2016.1).

##### Description of the search‐string

The search string is based on the PICO(s)‐model, and contains two concepts, of which we have developed two corresponding search facets: population characteristics and the intervention. The search string includes searches in title and abstract as well as subject terms and/or keywords for each facet. The subject terms in the facets will be selected according to the thesaurus or index of each database. Keywords will be supplied if the search technique provides additional results. Use of truncation and wildcards will be used to address English spelling variants.

##### Example of a search‐string

The search string below is developed to search ERIC through the EBSCO search interface and exemplifies the search facets as they will be searched:

**Table:** 

#	Query
S13	S4 AND S9 AND S12
S12	S10 OR S11
S11	adolescen* OR child* OR girl* OR boy* OR juvenil* OR kid* OR minors OR paediatric* OR pediatric* OR pre‐school* OR preschool* OR puber* OR pubescen* OR school* OR teen* OR toddler* OR underage* OR underage* OR youth* OR youngster* OR young OR student*
S10	DE ‘Children’ OR DE ‘African American Children’ OR DE ‘Latchkey Children’ OR DE ‘Migrant Children’ OR DE ‘Minority Group Children’ OR DE ‘Preadolescents’ OR DE ‘Young Children’ OR DE ‘Adolescents’ OR DE ‘Early Adolescents’ OR DE ‘Late Adolescents’ OR DE ‘Preadolescents’ OR DE ‘Secondary School Students’ OR DE ‘High School Students’ OR DE ‘Junior High School Students’ OR DE ‘Youth’ OR DE ‘Disadvantaged Youth’ OR DE ‘Out of School Youth’ OR DE ‘Rural Youth’ OR DE ‘Urban Youth’ OR DE ‘Student welfare’
S9	S5 OR S6 OR S7 OR S8
S8	(FRIENDS OR ‘Friends for Life’ OR ‘My Friends Youth’ OR ‘Fun Friends’) N6 program*
S7	prevent*
S6	DE ‘Health Promotion’ OR DE ‘Preventive Medicine’
S5	DE ‘Prevention’ OR DE ‘Dropout Prevention’
S4	S1 OR S2 OR S3
S3	SU (anxiety OR panic* OR phobia* OR phobic* OR sociophobi* OR socio‐phobi* OR GAD)
S2	TI (anxiety OR panic* OR phobia* OR phobic* OR sociophobi* OR socio‐phobi* OR GAD)
S1	DE ‘Anxiety Disorders’ OR DE ‘Anxiety’ OR DE ‘Separation Anxiety’ OR DE ‘Test Anxiety’

#### 3.2.2 Searching other resources

##### Hand‐Search

We will conduct a hand search of specific journals, to make sure that all relevant articles are found. The hand search will focus on editions published between 2021 and 2023 to secure recently unpublished articles which have not yet been indexed in the bibliographic databases. We will decide upon which journals to hand search based on the identified records from the electronic searches. The following are examples of specific journals which we may decide to hand search:

- British Journal of Clinical Psychology
- Behaviour Change
- Journal of Clinical Child and Adolescent Psychology
- Clinical Child Psychology and Psychiatry
- Journal of Primary Prevention
- Child and Adolescent Mental Health
- European Journal of Child and Adolescent Psychiatry
- Advances in School Mental Health Promotion

##### Grey literature searches

We will search for such references as dissertations, working papers and conference proceedings, reports, and EGM's or systematic reviews. Most of the resources searched may include multiple types of references, both published and unpublished. In general, there is a great amount of overlap between the types of references in the chosen resources. The resources are listed once under the category of literature we expect to be most prevalent in the resource, even though multiple types of unpublished/published literature might be identified in the resource. A final list of resources will be included in an appendix.

*Artificial Intelligence search for references on the Internet*

- Elicit.org

*Dissertations*

We will search the following resources for dissertations:

- Open Access Theses and Dissertations
- EBSCO Open Dissertations (EBSCO‐host)

*Working papers and conference proceedings*

We will search the following resources for working papers/conference proceedings:

- Google Scholar: https://scholar.google.com/
- Social Science Research Network: https://www.ssrn.com/index.cfm/en/
- Conference Proceedings Citation Index

For each resource, we will screen the first 100 hits.

*Evidence and gap maps and systematic reviews*

To locate any potential EGMs or systematic reviews, we will search the following resources:

- Campbell Systematic Reviews Journal: https://onlinelibrary.wiley.com/journal/18911803?af=R
- EPPI‐Centre publications: https://eppi.ioe.ac.uk/cms/Default.aspx?tabid=116
- PROSPERO: https://www.crd.york.ac.uk/prospero/
- Epistemonikos: https://www.epistemonikos.org/

If we identify relevant systematic reviews or EGMs during the search process, they will be used for citation‐tracking, to extract relevant references from the review.

*Trial registries*

- CENTRAL Trials Register within the Cochrane Library: https://www.cochranelibrary.com/central (includes ClinicalTrials.gov: https://clinicaltrials.gov/ and WHO International Clinical Trials Registry Platform: https://www.who.int/clinical-trials-registry-platform

### 3.3 Data collection and analysis

#### 3.3.1 Description of methods used in primary research

Randomised controlled trials are eligible, and we expect that most studies will be conducted with randomisation of participants. Studies conducted without randomisation of participants are eligible but are required to have a control group for inclusion in the review. Participants may be allocated by, for example, location differences, decision makers, policy rules or participant preferences. We expect that the primary studies demonstrate pretreatment group equivalence via matching, statistical controls, or evidence of equivalence on key risk variables and participant characteristics as outlined in the Section 3.3.4.

#### 3.3.2 Selection of studies

Under the supervision of review authors, two review team assistants will first independently screen titles and abstracts to exclude studies that are clearly irrelevant. Studies considered eligible by at least one assistant or studies were there is insufficient information in the title and abstract to judge eligibility, will be retrieved in full text. The full texts will then be screened independently by two review team assistants under the supervision of the review authors. Any disagreement of eligibility will be resolved by the review authors. Exclusion reasons for studies that otherwise might be expected to be eligible will be documented and presented in an appendix.

The study inclusion criteria will be piloted by the review authors and team assistants (see Supporting Information: Appendix 2). The overall search and screening process will be illustrated in a flow diagram. None of the review team members will be blind to the authors, institutions, or the journals responsible for the publication of the articles.

#### 3.3.3 Data extraction and management

Two review authors will independently code and extract data from all included studies. A coding sheet will be piloted on several studies and revised as necessary (see Supporting Information: Appendix 3). Disagreements will be resolved by consulting a third review author with extensive content and methods expertise. Disagreements resolved by a third reviewer will be reported. Data and information will be extracted on: available characteristics of participants, intervention characteristics and control conditions, research design, sample size, risk of bias and potential confounding factors, outcomes, and results. Extracted data will be stored electronically. Analysis will be conducted using RevMan5 and Stata software.

#### 3.3.4 Assessment of risk of bias in included studies

We will assess the risk of bias in randomised studies using Cochrane's revised risk of bias tool, ROB 2 (Higgins et al., 2019).

The tool is structured into five domains, each with a set of signalling questions to be answered for a specific outcome. The five domains cover all types of bias that can affect results of randomised trials.

The five domains for individually randomised trials are:

(1) bias arising from the randomisation process;
(2) bias due to deviations from intended interventions (separate signalling questions for effect of assignment and adhering to intervention);
(3) bias due to missing outcome data;
(4) bias in measurement of the outcome;
(5) bias in selection of the reported result.

For cluster‐randomised trials, an additional domain is included ([1b] Bias arising from identification or recruitment of individual participants within clusters). We will use the latest template for completion (currently it is the version of 15 March 2019 for individually randomised parallel‐group trials and 20 October 2016 for cluster randomised parallel‐group trials). In the cluster randomised template however, only the risk of bias due to deviation from the intended intervention (effect of assignment to intervention; intention to treat ITT) is present and the signalling question concerning the appropriateness of the analysis used to estimate the effect is missing. Therefore, for cluster randomised trials we will only use the signalling questions concerning the bias arising from identification or recruitment of individual participants within clusters from the template for cluster randomised parallel‐group trials; otherwise we will use the template and signalling questions for individually randomised parallel‐group trials.

We will assess the risk of bias in non‐randomised studies, using the model ROBINS –I, developed by members of the *Cochrane Bias Methods Group* and the *Cochrane Non‐Randomised Studies Methods Group* (Sterne et al., 2016a). We will use the latest template for completion (currently it is the version of 19 September 2016).

The ROBINS‐I tool is based on the Cochrane RoB tool for randomised trials, which was launched in 2008 and modified in 2011 (Higgins et al., 2011).

The ROBINS‐I tool covers seven domains (each with a set of signalling questions to be answered for a specific outcome) through which bias might be introduced into non‐randomised studies:

(1) bias due to confounding;
(2) bias in selection of participants;
(3) bias in classification of interventions;
(4) bias due to deviations from intended interventions;
(5) bias due to missing outcome data;
(6) bias in measurement of the outcome;
(7) bias in selection of the reported result.

The first two domains address issues before the start of the interventions and the third domain addresses classification of the interventions themselves. The last four domains address issues after the start of interventions and there is substantial overlap for these four domains between bias in randomised studies and bias in non‐randomised studies trials (although signalling questions are somewhat different in several places, see Sterne et al., 2016b and Higgins et al., 2019).

Randomised study outcomes are rated on a ‘Low/Some concerns/High’ scale on each domain; whereas non‐randomised study outcomes are rated on a ‘Low/Moderate/Serious/Critical/No Information’ scale on each domain. The level ‘Critical’ means: the study (outcome) is too problematic in this domain to provide any useful evidence on the effects of intervention, and it is excluded from the data synthesis. The same critical level of risk of bias (excluding the result from the data synthesis) is not directly present in the RoB 2 tool, according to the guidance to the tool (Higgins et al., 2019).

In the case of an RCT, where there is evidence that the randomisation has gone wrong or is no longer valid, we will assess the risk of bias of the outcome measures using ROBINS‐I instead of ROB 2. Examples of reasons for assessing RCTs using the ROBINS‐I tool may include studies showing large and systematic differences between treatment conditions while not explaining the randomisation procedure adequately suggesting that there was a problem with the randomisation process; studies with large scale differential attrition between conditions in the sample used to estimate the effects; or studies selectively reporting results for some part of the sample or for only some of the measured outcomes. In such cases, differences between the treatment and control conditions are likely systematically related to other factors than the intervention and the random assignment is, on its own, unlikely to produce unbiased estimates of the intervention effects. Therefore, as ROBINS‐I allow for an assessment of for example confounding, we believe it is more appropriate to assess effect sizes from studies with a compromised randomisation using ROBINS‐I than ROB 2. If so, we will report this decision as part of the risk of bias assessment of the outcome measure in question. As other effect sizes assessed with ROBINS‐I, these effect sizes may receive a ‘Critical’ rating and thus be excluded from the data synthesis.

We will stop the assessment of a non‐randomised study outcome as soon as one domain in the ROBINS‐I is judged as ‘Critical’.

‘Serious’ risk of bias in multiple domains in the ROBINS‐I assessment tool may lead to a decision of an overall judgement of ‘Critical’ risk of bias for that outcome, and it will be excluded from the data synthesis.

##### Confounding

An important part of the risk of bias assessment of non‐randomised studies is consideration of how the studies deal with confounding factors. Systematic baseline differences between groups can compromise comparability between groups. Baseline differences can be observable (e.g., age and gender) and unobservable (to the researcher; e.g., motivation and ‘ability’). There is no single non‐randomised study design that always solves the selection problem. Different designs represent different approaches to dealing with selection problems under different assumptions, and consequently require different types of data. There can be particularly great variations in how different designs deal with selection on unobservables. The ‘adequate’ method depends on the model generating participation, that is, assumptions about the nature of the process by which participants are selected into a programme.

As there is no universal correct way to construct counterfactuals for non‐randomised designs, we will look for evidence that identification is achieved, and that the authors of the primary studies justify their choice of method in a convincing manner by discussing the assumption(s) leading to identification (the assumption(s) that make it possible to identify the counterfactual). Preferably the authors should make an effort to justify their choice of method and convince the reader that the only difference between a treated individual and a non‐treated individual is the treatment. The judgement is reflected in the assessment of the confounder unobservables in the list of confounders considered important at the outset (see Supporting Information: Appendix 4).

In addition to unobservables, we have identified the following observable confounding factors to be most relevant: age, gender, socio‐economic status (SES) and anxiety symptoms at baseline. In each study, we will assess whether these indicators have been considered, and in addition we will assess other factors likely to be a source of confounding within the individual included studies.

##### Importance of pre‐specified confounding factors

The motivation for focusing on age, gender, SES and anxiety symptoms at baseline is given below.

The prevalence of different types of psychological problems, coping skills, cognitive and emotional ability vary throughout a child's development through puberty and into adulthood (Cole et al., 2005), and therefore we consider age to be a potential confounding factor.

Furthermore, there are substantial (although inconsistent) gender differences in fear reporting, coping and risk of different types of anxiety disorders, which is why we also include gender as a potential confounding factor (Dalsgaard et al., 2020; Hampel & Petermann, 2005; McLean & Anderson, 2009).

Low childhood SES, in particular financial hardship, is associated with increased exposure to a range of childhood adversities (CAs) such as parental psychopathology, maltreatment, and family violence. Exposure to CAs as well as financial hardship have been associated with increased levels of anxiety in children and onset of anxiety disorders in childhood (Green et al., 2010; McLaughlin et al., 2011).

Finally, pre‐treatment group equivalence of anxiety symptoms is indisputable an important confounder. Therefore, the accuracy of the estimated effects of FRIENDS programmes will depend crucially on how well pre‐treatment anxiety symptoms are controlled for.

##### Effect of primary interest and important co‐interventions

We are mainly interested in the effect of starting and adhering to the intended intervention, that is, the treatment on the treated effect. The risk of bias assessments will therefore be in relation to this specific effect.

The risk of bias assessments of both randomised trials and non‐randomised studies will consider adherence and differences in additional interventions (‘co‐interventions’) between intervention groups. Relevant co‐interventions are those that individuals might receive with or after starting the intervention of interest and that are both related to the intervention received and prognostic for the outcome of interest. Important co‐interventions we will consider are any kind of mental health treatments delivered on an individual basis.

##### Assessment

At least two review authors will independently assess the risk of bias for each relevant outcome from the included studies. Any disagreements will be resolved by a third reviewer with content and statistical expertise and will be reported. We will report the risk of bias assessment in risk of bias tables for each included study outcome in the completed review.

#### 3.3.5 Measures of treatment effect

##### Continuous outcomes

For continuous outcomes, effects sizes with 95% confidence intervals will be calculated, where means and standard deviations are available. If means and standard deviations are not available, we will calculate standardised mean differences (SMDs) from *F*‐ratios, *t*‐values, chi‐squared values and correlation coefficients, where available, using the methods suggested by Lipsey and Wilson (2001). If not enough information is yielded, the review authors will request this information from the principal investigators. Hedges' *g* will be used for estimating SMDs. Any measures of anxiety and self‐esteem outcomes, are examples of relevant continuous outcomes in this review.

##### Dichotomous outcomes

For dichotomous outcomes, we will calculate odds ratios with 95% confidence intervals. Prevalence of anxiety diagnosis is an example of a relevant dichotomous outcome in this review.

There are statistical approaches available to re‐express dichotomous and continuous data to be pooled together (Sánchez‐Meca et al., 2003). To calculate common metric odds ratios will be converted to SMD effect sizes using the Cox transformation. We will only transform dichotomous effect sizes to SMD if appropriate, for example, as may be the case if a study reports anxiety symptoms as a dichotomous outcome.

When effect sizes cannot be pooled, study‐level effects will be reported in as much detail as possible. Software for storing data and statistical analyses will be RevMan, Excel, R and Stata 10.0.

#### 3.3.6 Unit of analysis issues

Errors in statistical analysis can occur when the unit of allocation differs from the unit of analysis. In cluster randomised trials, participants are randomised to treatment and control groups in clusters, either when data from multiple participants in a setting are included (creating a cluster within school or community setting), or when participants are randomised by treatment locality or school. Non‐randomised studies may also include clustered assignment of treatment. Effect sizes and standard errors from such studies may be biased if the unit‐of‐analysis is the individual and an appropriate cluster adjustment is not used (Higgins & Green, 2011).

A study design where participants are individually allocated to treatment, but the treatment is delivered in a group setting, are known as *individually randomised group treatment* trials (Pals et al., 2008). The analysis in such a study design must also correct for the fact that dependencies may arise between individuals that happen to receive the intervention in the same group.

If possible, we will adjust effect sizes individually using the methods suggested by Hedges (2007b) and information about the intra‐cluster correlation coefficient (ICC), realised cluster sizes, and/or estimates of the within and between variances of clusters. If it is not possible to obtain this information, we will adjust effect sizes using estimates from the literature (we will search for estimates of relevant ICC's), and assume equal cluster sizes. To calculate an average cluster size, we will divide the total sample size in a study by the number of clusters.

We will perform analyses separated by the time points suggested in Section 3.1.5.

#### 3.3.7 Criteria for determination of independent findings

To determine the independence of results in included studies, we will consider whether individuals may have undergone multiple interventions, whether there were multiple treatment groups, whether several studies are based on the same data source and whether studies report multiple conceptually similar outcomes.

##### Multiple interventions groups and multiple interventions per individuals

Studies with multiple intervention groups with different individuals will be included in this review, although only intervention and control groups that meet the eligibility criteria will be used in the data synthesis. To avoid problems with dependence between effect sizes we will apply robust standard errors (Hedges et al., 2010) and use the small sample adjustment to the estimator itself (Tipton, 2015). We will use the results in Tanner‐Smith and Tipton (2014) and Tipton (2015) to evaluate if there are enough studies for this method to consistently estimate the standard errors. See Section 3.3.11 below for more details about the data synthesis.

If there are not enough studies, we will use a synthetic effect size (the average) to avoid dependence between effect sizes. This method provides an unbiased estimate of the mean effect size parameter but overestimates the standard error. Random effects models applied when synthetic effect sizes are involved actually perform better in terms of standard errors than do fixed effects models (Hedges, 2007a). However, tests of heterogeneity when synthetic effect sizes are included are rejected less often than nominal.

If pooling is not appropriate (e.g., the multiple interventions and/or control groups include the same individuals), only one intervention group will be coded and compared to the control group to avoid overlapping samples. The choice of which estimate to include will be based on our risk of bias assessment. We will choose the estimate that we judge to have the least risk of bias (primarily, Confounding bias and in case of equal scoring the Missing outcome data domain will be used).

##### Multiple studies using the same sample of data

In some cases, several studies may have used the same sample of data or some studies may have used only a subset of a sample used in another study. We will review all such studies, but in the meta‐analysis we will only include one estimate of the effect from each sample of data. This will be done to avoid dependencies between the ‘observations’ (i.e., the estimates of the effect) in the meta‐analysis. The choice of which estimate to include will be based on our risk of bias assessment of the studies. We will choose the estimate from the study that we judge to have the least risk of bias (primarily, Confounding bias). If two (or more) studies are judged to have the same risk of bias and one of the studies (or more) uses a subset of a sample used in another study (or studies) we will include the study using the full set of participants.

##### Multiple time points

When the results are measured at multiple time points, each outcome at each time point will be analysed in a separate meta‐analysis with other comparable studies taking measurements at a similar time point. As a general guideline, these will be grouped together as stated in Section 3.1.5. However, should the studies provide viable reasons for an adjusted choice of relevant and meaningful duration intervals for the analysis of outcomes, we will adjust the grouping.

##### Multiple conceptually similar outcomes

Meta‐analysis of outcomes will be conducted on each metric (as outlined in Section 3.1.4) separately. If there are multiple estimates of effects regarding the same/similar outcome (e.g., anxiety symptoms measured with both the SCAS and the RCMAS measures), we will extract (and report) all outcomes, but in the meta‐analysis we will include one measure. We will include the most validated, best recognised, or most frequently used measures in the analysis. We will prioritise broad measures of anxiety symptoms (e.g., SCAS, SCARED, MASC, RCMAS, RCADS, CBCL‐A), rather than disorder‐specific symptom measures (e.g., SPAI‐C, SAS‐A, TASC).

Further, these scales could be self‐report or completed by a parent or teacher. Multiple reporters are often used; we will analyse reduction in anxiety symptoms separately for (1) self‐reported and (2) parent‐reported or teacher, or both.

Concerning the secondary outcome: If multiple self‐esteem measures/reporters are reported, we include the most validated, best recognised, or most frequently used measures in the analysis.

#### 3.3.8 Dealing with missing data

Missing data and attrition rates will be assessed in the included studies; see Section 3.3.4. Where studies have missing summary data, such as missing standard deviations, the review authors will request this information from the principal investigators. If no information is yielded, we will derive these where possible from *F*‐ratios, *t*‐values, chi‐squared values and correlation coefficients using the methods suggested by Lipsey and Wilson (2001). If missing summary data cannot be derived, the study results will be reported in as much detail as possible.

#### 3.3.9 Assessment of heterogeneity

Heterogeneity amongst primary outcome studies will be assessed with chi‐squared (*Q*) test, and the *I*‐squared, and *τ*‐squared statistics (Higgins et al., 2003). Any interpretation of the chi‐squared test will be made cautiously on account of its low statistical power.

#### 3.3.10 Assessment of reporting biases

Reporting bias refers to both publication bias and selective reporting of outcome data and results. Here, we state how we will assess publication bias.

We will use funnel plots for information about possible publication bias if we find sufficient studies (Higgins & Green, 2011). However, asymmetric funnel plots are not necessarily caused by publication bias (and publication bias does not necessarily cause asymmetry in a funnel plot). In general, asymmetry is a sign of small‐study effects, of which there can be many causes beside publication bias (Sterne et al., 2005).

Instead of trying to interpret the funnel plots as direct evidence of publication bias, or the lack thereof, we will perform sensitivity analyses for publication bias in meta‐analyses as suggested by Mathur and VanderWeele (2020). This method gives a value of how large ratios of publication probabilities (i.e., the likelihood of affirmative results to be published relative to non‐affirmative results) would have to be to alter the results and therefore indicate how robust the meta‐analysis is to publication bias.

#### 3.3.11 Data synthesis

The proposed project will follow standard procedures for conducting systematic reviews using meta‐analysis techniques.

All follow‐up durations reported in the primary studies will be recorded, and we will do separate analyses for post, short‐term, medium term and long‐term outcomes.

The overall data synthesis will be conducted where effect sizes are available or can be calculated, and where studies are similar in terms of the outcome measured. Meta‐analysis of outcomes will be conducted on each metric (as outlined in Section 3.1.4) separately.

As different computational methods may produce effect sizes that are not comparable, we will be transparent about all methods used in the primary studies (research design and statistical analysis strategies) and use caution when synthesising effect sizes. Special caution will be taken concerning studies using regression discontinuity designs (RDD) to estimate the treatment effect. In sharp RDDs, a threshold of a (non‐manipulable) forcing/running variable determines which students receive a treatment and which do not, that is, the design is similar to a RCT in the sense that the random sequence determining treatment assignment can be seen as a running variable (Lee & Lemieux, 2010). In contrast, in ‘fuzzy’ RDDs, being on one side of a threshold is a special type of IV only makes it more likely that a student ends up in the treatment or control group, and the threshold is used as an instrument to estimate local average treatment effects (Angrist & Pischke, 2009; Imbens & Lemieux, 2008). That is, fuzzy RDD is a form of IV analysis, which we will exclude due to the comparability issues mentioned earlier. Sharp RDDs will be included, but, as the effects may be estimated on a very ‘local’ sample close to a threshold, may be subject to a separate analysis depending on the comparability to samples from other studies. We will in any case check the sensitivity of our results to the inclusion of RDD studies. In addition, we will discuss the limitation in generalisation of results obtained from these types of studies.

When the effect sizes used in the data synthesis are odds ratios, they will be log transformed before being analysed. The reason is that ratio summary statistics all have the common feature that the lowest value that they can take is 0, that the value 1 corresponds with no intervention effect, and the highest value that an odds ratio can ever take is infinity. This number scale is not symmetric. The log transformation makes the scale symmetric: the log of 0 is minus infinity, the log of 1 is zero, and the log of infinity is infinity.

Studies that have been coded with a Critical risk of bias will not be included in the data synthesis.

As the intervention deals with diverse populations of participants (from different countries, facing different life circumstances, etc.), and we therefore expect heterogeneity amongst primary study outcomes, all analyses of the overall effect will be inverse variance weighted using random effects statistical models that incorporate both the sampling variance and between study variance components into the study level weights. Random effects weighted mean effect sizes will be calculated using 95% confidence intervals, and we will provide a graphical display (forest plot) of effect sizes. Graphical displays for meta‐analysis performed on ratio scales sometimes use a log scale, as the confidence intervals then appear symmetric. This is however not the case for the software Revman 5 which we plan to use in this review (If we apply robust variance estimation [RVE], the analysis will be conducted in Stata or R as RVE is not implemented in Revman 5). The graphical displays using odds ratios and the mean effect size will be reported as an odds ratio. Heterogeneity amongst primary outcome studies will be assessed with chi‐squared (*Q*) test, and the *I*‐squared, and *τ*‐squared statistics (Higgins et al., 2003). Any interpretation of the chi‐squared test will be made cautiously on account of its low statistical power.

In addition to 95% confidence intervals we will report 95% prediction intervals.

For subsequent analyses of moderator variables that may contribute to systematic variations, we will use the mixed‐effects regression model if there are a sufficient number of studies. This model is appropriate if a predictor explaining some between‐studies variation is available, but there is a need to account for the remaining uncertainty (Hedges & Piggott, 2004; Konstantopoulos, 2006).

Studies may provide results separated by, for example, age and/or gender. We will include results for all age and gender groups. To take into account the dependence between such multiple effect sizes from the same study, we will apply RVE approach (Hedges et al., 2010). An important feature of this analysis is that the results are valid regardless of the weights used. For efficiency purposes, we will calculate the weights using a method proposed by Hedges et al. (2010). This method assumes a simple random‐effects model in which study average effect sizes vary across studies (*τ* ^2^) and the effect sizes within each study are equi correlated (*ρ*). The method is approximately efficient, since it uses approximate inverse‐variance weights: they are approximate given that *ρ* is, in fact, unknown and the correlation structure may be more complex. We will calculate weights using estimates of *τ* ^2^, setting *ρ* = 0.80 and conduct sensitivity tests using a variety of *ρ* values; to assess if the general results and estimates of the heterogeneity is robust to the choice of *ρ*. We will use the small sample adjustment to the residuals used in RVE as proposed by Bell and McCaffrey (2002) and extended by McCaffrey et al. (2001) and by Tipton (2015). We will use the Satterthwaite degrees of freedom (Satterthwaite, 1946) for tests as proposed by Bell and McCaffrey (2002) and extended by Tipton (2015). We will use the guidelines provided in Tanner‐Smith and Tipton (2014) to evaluate if there are enough studies for this method to consistently estimate the standard errors.

If there is not a sufficient number of studies to use RVE we will conduct a data synthesis where we use a synthetic effect size (the average) to avoid dependence between effect sizes.

#### 3.3.12 Subgroup analysis and investigation of heterogeneity

We will investigate the following factors with the aim of explaining potential observed heterogeneity:

Type of programme, that is, whether it is a universal, indicated or selective intervention; and the three different age‐appropriate programmes: ‘Fun Friends’ (4–7 year olds), ‘Friends for Life’ (8–11 year olds) and ‘My Friends Youth’ (12–16 year olds), or the corresponding before 2005 versions ‘FRIENDS *for Children*’ (7–11 year‐olds), and ‘FRIENDS *for Youth*’ (12–16 year‐olds). Other study‐level summaries of participant characteristics (e.g., studies considering a specific gender or studies where separate effects for girls/boys are available) and SES indicator (e.g., studies considering a specific SES indicator or studies where separate effects for low/high socioeconomic status are available). In addition, we will investigate other programme characteristics such as type of provider (lay/teacher or mental health provider), country of implementation (Australia/other countries) and implementation issues in relation to the booster sessions and parent sessions (implemented, partly implemented or not at all).

If the number of included studies is sufficient and given there is variation in the covariates (age, gender, SES and programme characteristics), we will perform moderator analyses (multiple meta‐regression using the mixed model) to explore how observed variables are related to heterogeneity.

If there are a sufficient number of studies, we will apply the RVE approach and use approximately inverse variance weights calculated using a method proposed by Hedges et al. (2010). This technique calculates standard errors using an empirical estimate of the variance: it does not require any assumptions regarding the distribution of the effect size estimates. The assumptions that are required to meet the regularity conditions are minimal and generally met in practice. This more robust technique is beneficial because it takes into account the possible correlation between effect sizes separated by the covariates within the same study (e.g., age or gender separated effects) and allows all the effect size estimates to be included in meta‐regression. We will calculate weights using estimates of *τ* ^2^, setting *ρ* = 0.80 and conduct sensitivity tests using a variety of *ρ* values; to assess if the general results are robust to the choice of *ρ*. We will use the small sample adjustment to the residuals used in RVE and the Satterthwaite degrees of freedom (Satterthwaite, 1946) for tests (Tipton, 2015). The results in Tipton (2015) suggests that the degrees of freedom depend on not only the number of studies but also on the type of covariates included in the meta‐regression. The degrees of freedom can be small, even when the number of studies is large if a covariate is highly unbalanced or a covariate with very high leverage is included, The degrees of freedom will vary from coefficient to coefficient. The corrections to the degrees of freedom enable us to assess when the RVE method performs well. As suggested by Tanner‐Smith and Tipton (2014) and Tipton (2015) if the degrees of freedom are smaller than four, the RVE results should not be trusted.

We will report 95% confidence intervals for regression parameters. We will estimate the correlations between the covariates and consider the possibility of confounding. Conclusions from meta‐regression analysis will be cautiously drawn and will not solely be based on significance tests. The magnitude of the coefficients and width of the confidence intervals will be taken into account as well. Otherwise, single factor subgroup analysis will be performed. The assessment of any difference between subgroups will be based on 95% confidence intervals. Interpretation of relationships will be cautious, as they are based on subdivision of studies and indirect comparisons.

In general, the strength of inference regarding differences in treatment effects amongst subgroups is controversial. However, making inferences about different effect sizes amongst subgroups on the basis of between‐study differences entails a higher risk compared to inferences made on the basis of within study differences (see Schandelmaier et al., 2020). We will therefore use within study differences where possible.

We will also consider the degree of consistence of differences, as making inferences about different effect sizes amongst subgroups entails a higher risk when the difference is not consistent within the studies (Schandelmaier et al., 2020).

#### 3.3.13 Sensitivity analysis

Sensitivity analysis will be carried out by restricting the meta‐analysis to a subset of all studies included in the original meta‐analysis and will be used to evaluate whether the pooled effect sizes are robust across components of risk of bias. We will consider sensitivity analysis for each domain of the risk of bias checklists and restrict the analysis to studies with a low risk of bias.

Sensitivity analyses with regard to research design and statistical analysis strategies in the primary studies will be an important element of the analysis to ensure that different methods produce consistent results.

#### 3.3.14 Treatment of qualitative research

We do not plan to include qualitative research.

#### 3.3.15 Summary of findings and assessment of the certainty of the evidence

The GRADE (Grades of Recommendation, Assessment, Development, and Evaluation) system will be used to assess the certainty of the body of evidence as it relates to the studies that contribute data to the meta‐analyses for the prespecified outcomes (Guyatt et al., 2008). The system classifies certainty of evidence as high, moderate, low, or very low (Guyatt et al., 2013). Five of the eight criteria proposed in the GRADE method have the potential to decrease one's confidence in the correctness of the effect estimates: risk of bias, inconsistency of results across studies, indirectness of evidence, imprecision, and publication bias. Three further criteria are proposed that have the potential to increase this confidence: a large magnitude of effect with no plausible confounders, a dose–response gradient, and a conclusion that all plausible residual confounding would further support inferences regarding treatment effect. GRADE proposes these three criteria should be considered particularly in observational studies. (Guyatt et al., 2011). We will justify all decisions to downgrade or upgrade the certainty of outcomes, and make comments to aid readers' understanding of the review where necessary.

The outcomes will be graded as follows.

- High certainty: further research is very unlikely to change our confidence in the effect estimate.
- Moderate certainty: further research is likely to have an important impact on our confidence in the effect estimate and may change the estimate.
- Low certainty: further research is very likely to have an important impact on our confidence in the effect estimate and may change the estimate.
- Very low certainty: we are very uncertain about the effect estimate.

We will use the GRADEpro GDT software (available at https://gdt.gradepro.org/app/) to produce a summary of findings table, presenting the overall quality of the body of evidence according to GRADE criteria for the mental health outcomes.

## CONTRIBUTIONS OF AUTHORS

Please give brief description of content and methodological expertise within the review team. The recommended optimal review team composition includes at least one person on the review team who has content expertise, at least one person who has methodological expertise and at least one person who has statistical expertise. It is also recommended to have one person with information retrieval expertise.

Who is responsible for the below areas? Please list their names:

- Content: Trine Filges, Geir Smedslund, Tine L. Mundbjerg Eriksen
- Systematic review methods: Trine Filges, Geir Smedslund
- Statistical analysis: Trine Filges, Geir Smedslund, Tine L. Mundbjerg Eriksen
- Information retrieval: Kirsten Birkefoss

## DECLARATIONS OF INTEREST

Please declare any potential conflicts of interest. For example, have any of the authors been involved in the development of relevant interventions, primary research, or prior published reviews on the topic?

## SOURCES OF SUPPORT

**Internal sources**

- VIVE Campbell, Denmark
**External sources**
- New Source of support, Other

## Supporting information

Supporting information.
