# Supplementary material for: PROTOCOL: The FRIENDS preventive programme for reducing anxiety symptoms in children and adolescents: A systematic review
Source: Campbell Syst Rev. 2023 Dec 15;19(4):e1374. doi: 10.1002/cl2.1374 (PMC10723782; doi:10.1002/cl2.1374)
Supplement: Supplementary file 1 — Supporting information. [file CL2-19-e1374-s001.docx]

# Appendices

## 1 Justification of exclusion of studies using an instrumental variable (IV) approach

The following is based on [Angrist and Pischke, 2009](#REF-Angrist-and-Pischke_x002c_-2009); [Heckman et al., 2006](#REF-Heckman-et-al._x002c_-2006) and [Heckman and Urzúa ,2010](#REF-Heckman-and-Urz_x00fa_a-_x002c_2010).

Studies using instrument variables (IV) for causal inference in non-randomised studies will not be included as the interpretation of IV estimates is challenging. IV only provides an estimate for a specific group namely, people whose behaviour change due to changes in the particular instrument used. It is not informative about effects on never-takers and always-takers because the instrument does not affect their treatment status. The estimated effect is thus applicable only to the subpopulation whose treatment status is affected by the instrument. As a consequence, the effects differ for different IVs and care has to be taken as to whether they provide useful information. The effect is interesting when the instrument it is based on is interesting in the sense that it corresponds to a policy instrument of interest. Further, if those that are affected by the instrument are not affected in the same way the IV estimate is an average of the impacts of changing treatment status in both directions, and cannot be interpreted as a treatment effect. To turn the IV estimate into a LATE requires a monotonicity assumption. The movements induced by the instrument go in one direction only, from no treatment to treatment. The IV estimate, interpreted as a LATE, is only applicable to the complier population, those that are affected by the instrument in the ‘right way’. It is not possible to characterise the complier population as an observation’s subpopulation cannot be determined and defiers do not exist by assumption.

In the binary-treatment–binary-instrument context, the IV estimate can, given monotonicity, be interpreted as a LATE; i.e. the average treatment effect for the subpopulation of compliers. If treatment or instruments are not binary, interpretation becomes more complicated. In the binary-treatment–multivalued-instrument (ordered to take values from 0 to *J*) context, the IV estimate, given monotonicity, is a weighted average of pairwise LATE parameters (comparing subgroup *j* with subgroup *j*−1). The IV estimate can thus be interpreted as the weighted average of average treatment effects in each of the *J* subgroups of compliers. In the multivalued-treatment (ordered to take values from 0 to *T*) – multivalued-instrument (ordered to take values from 0 to *J*) context, the IV estimate for *each pair of instrument values*, given monotonicity, is a weighted average of the effects from going from *t*-1 to *t* for persons induced by the change in the value of the instrument to move from any level below *t* to the level *t* or any level above. Persons can be counted multiple times in forming the weights.

## 2 First and second level screening

First level screening is on the basis of titles and abstracts. Second level is on the basis of full text

Reference id. No. :

Reviewers initials:

Source:

Year of publication:

Country/countries of origin:

Author(s):

The study will be excluded if one or more of the answers to question 1-3 are ‘No’. If the answers to question 1 to 3 are ‘Yes’ or ‘Uncertain’, then the full text of the study will be retrieved for second level eligibility. All unanswered questions need to be posed again on the basis of the full text. If not enough information is available, or if the study is unclear, the author of the study will be contacted if possible.

**Screening questions:**

1. Does the study focus on one of the three age-appropriate preventive anxiety programmes FRIENDS (Fun FRIENDS (4 – 7 years), FRIENDS for life (8 – 11 years), My FRIENDS Youth (12 – 16 years))?

Yes - include

No – if no then stop here and exclude

Uncertain - include

Question 1 guidance:

Note that these three age-appropriate programmes and their titles are the current versions of the FRIENDS programme. Prior to 2005, they were titled: FRIENDS *for Children* 7–11 years, and FRIENDS *for Youth* 12–16 years and Fun FRIENDS was added.

The intervention in this review is participation in any type of preventive provision.

Specifically, universal prevention programmes are applied to the general population, without focusing on the risk status. Selective programmes target those (individuals or groups) who are identified as exhibiting an elevated risk for developing a disorder based on established risk factors (for example socio economic status), and indicated programs target those (individuals) who exhibit problematic behaviors predicting a high level of risk or initial symptoms of a disorder but who do not yet meet criteria for the disorder.

All types of preventive programmes are eligible whereas treatment programmes (for those with a diagnosis and in need of treatment) are not eligible.

All types of providers (e.g. teachers, mental health providers) and all types of settings (e.g. school based, community based) are eligible.

2. Are the participants children and adolescents aged 4 to 16 years who do not meet diagnostic criteria for an anxiety disorder diagnosis?

Yes - include

No – if no then stop here and exclude

Uncertain - include

Question 2 guidance:

We anticipate that the three age-appropriate programmes will be provided to children and adolescents in the corresponding age groups; i.e. ‘Fun Friends’ to 4-7 year olds, ‘Friends for Life’ to 8-11 year olds and ‘My Friends Youth’ to 12-16 year olds. If studies including participants out-of-age range are located they will be eligible if the majority of participants are within the age range corresponding to the particular programme or results for a discrete age group within the eligible range is provided. If studies include a mix of children and adolescents with and without a clinically diagnosed anxiety disorder, we will include them if the majority of participants are not diagnosed or results of the eligible subgroup (not diagnosed) is provided.

3. Is the report/article a quantitative evaluation study with a comparison condition?

Yes - include

No – if no then stop here and exclude

Uncertain - include

Question 3 guidance:

We are only interested in primary quantitative studies with a comparison group, where the authors have analysed the data. We are not interested in theoretical papers on the topic or surveys/reviews of studies of the topic. (This question may be difficult to answer on the base of titles and abstracts alone.)

## 3 Data extraction

| **Names of author(s)** |
| --- |
| **Title** |
| **Language** |
| **Journal** |
| **Year** |
| **Country** |
| **Programme type: ‘ Friends’ to 4-7 year olds, ‘Friends for Life’ to 8-11 year olds and ‘My Friends Youth’ (**list them all if more than one and note that prior to 2005 the programmes were named FRIENDS *for Children* 7–11 years, and FRIENDS *for Youth* 12–16 years), universal/indicated/selective, factors used for identifying eligible participants in case of selective or indicated intervention |
| **Participant characteristic** (age/grade, gender, ethnicity, SES, pre test) |
| **Intervention characteristics** (setting: school/clinical/community/other, provider: teacher/professional mental health provider/other, group size, number and timing of parent sessions) |
| **Duration (**number of weeks, months, there should be 10 one hour lessons plus two follow-up booster sessions to be completed after one month and three months, respectively**)** |
| **Intensity (**number of hours per week**)** |
| **Integrity of intervention** (the degree to which specified procedures or components of the intervention were implemented as planned) |
| **Time period covered by analysis** (divide into intervention and follow up) |
| **Control group** (wait-list, TAU, other intervention) |

**Outcome measures**

Instructions: Please enter outcome measures in the order in which they are described in the report. Note that a single outcome measure can be completed by multiple sources and at multiple points in time (data from specific sources and time-points will be entered later).

| # | Outcome  & measure | Reliability & Validity | Format | Direction | Pg# & notes |
| --- | --- | --- | --- | --- | --- |
| 1 |  | Info from:  Other samples  This sample  Unclear  Info provided: | Dichotomy  Continuous | High score or event is  Positive  Negative  Can’t tell |  |

* Repeat as needed

**OUT COME DATA**

**DICHOTOMOUS OUTCOME DATA**

| OUTCOME | TIME POINT (s) (record exact time from participation, there may be more than one, record them all) | SOURCE | VALID Ns | CASES | NON-CASES | STATISTICS | Pg. # & NOTES |
| --- | --- | --- | --- | --- | --- | --- | --- |
|  |  | Questionnaire  Admin data  Other (specify)  Unclear | Participation | Participation | Participation | RR (risk ratio)  OR (odds ratio)  SE (standard error)  95% CI  DF  P- value (enter exact p value if available)  Chi2  Other |  |
|  |  |  |  |  |  |  |  |
|  |  |  | Comparison | Comparison | Comparison |  |  |
|  |  |  |  |  |  |  |  |

*Repeat as needed

**CONTINUOUS OUTCOME DATA**

| OUTCOME | TIME POINT (s) (record exact time from participation, there may be more than one, record them all) | SOURCE  (specify) | VALID Ns | Means | SDs | STATISTICS | Pg. # & NOTES |
| --- | --- | --- | --- | --- | --- | --- | --- |
|  |  | Questionnaire  Admin data  Other (specify)  Unclear | Participation | Participation | Participation | P  t  F  Df  ES  Other |  |
|  |  |  |  |  |  |  |  |
|  |  |  | Comparison | Comparison | Comparison |  |  |
|  |  |  |  |  |  |  |  |

*Repeat as needed

## 4 User guide for unobservables

Systematic baseline differences between groups can compromise comparability between groups. Baseline differences can be observable (e.g. age and gender) and unobservable (to the researcher; e.g. motivation and ‘ability’). There is no single non-randomised study design that always solves the selection problem. Different designs solve the selection problem under different assumptions and require different types of data. Especially how different designs deal with selection on unobservables varies. The “right” method depends on the model generating participation, i.e. assumptions about the nature of the process by which participants are selected into a programme.

As there is no universal correct way to construct counterfactuals we will assess the extent to which the identifying assumptions (the assumption that makes it possible to identify the counterfactual) are explained and discussed (preferably the authors should make an effort to justify their choice of method). We will look for evidence that authors using e.g. (this is NOT an exhaustive list):

**Natural experiments:**

Discuss whether they face a truly random allocation of participants and that there is no change of behaviour in anticipation of e.g. policy rules.

**Matching (including propensity scores):**

Explain and discuss the assumption that there is no selection on unobservables, only selection on observables.

**(Multivariate, multiple) Regression:**

Explain and discuss the assumption that there is no selection on unobservables, only selection on observables. Further, discuss the extent to which they compare comparable people.

**Regression Discontinuity (RD):**

Explain and discuss the assumption that there is a (strict!) RD treatment rule. It must not be changeable by the agent in an effort to obtain or avoid treatment. Continuity in the expected impact at the discontinuity is required.

**Difference-in-difference (Treatment-control-before-after):**

Explain and discuss the assumption that the trends in treatment and control groups would have been parallel, had the treatment not occurred.
